# Effects of combination of obesity, diabetes, and hypoxia on inflammatory regulating genes and cytokines in rat pancreatic tissues and serum

**DOI:** 10.7717/peerj.13990

**Published:** 2022-10-04

**Authors:** Sarah Albogami, Aziza Hassan, Sekena H. Abdel-Aziem, Saqer Alotaibi, Fayez Althobaiti, Ahmed El-Shehawi, Alaa Alnefaie, Reem Abdulla Alhamed

**Affiliations:** 1 Department of Biotechnology, College of Science, Taif University, Taif, Saudi Arabia; 2 High Altitude Research Center, Taif University, Taif, Saudi Arabia; 3 Cell Biology Department, Genetic Engineering and Biotechnology Division, National Research Centre, Cairo, Egypt; 4 Department of Medical Services, King Faisal Medical Complex, Taif, Saudi Arabia

**Keywords:** Obesity, Diabetes, Hypoxia, Inflammatory, Pancreatic tissues, Serum, Genes, Cytokines

## Abstract

**Background:**

Obesity and diabetes are becoming increasingly prevalent around the world. Inflammation, oxidative stress, insulin resistance, and glucose intolerance are linked to both obesity and type 2 diabetes, and these disorders are becoming major public health issues globally.

**Methods:**

This study evaluated the effects of obesity, diabetes, and hypoxia on the levels of pro- and anti-inflammatory cytokines in rats. We divided 120 Wistar rats in two groups, male and female, each including six subgroups: control (CTRL), obese (high-fat diet (HFD)), diabetic (streptozotocin (STZ)-treated), hypoxic (HYX), obese + diabetic (HFD/STZ), and obese + diabetic + hypoxic (HFD/STZ/HYX). We examined the levels of tumor necrosis factor-*α* (TNF-*α*), interleukin (IL)-6, IL10, and leptin in pancreatic tissues and serum.

**Results:**

No significant difference was observed in serum levels of cholesterol, triglycerides, and low-density lipoprotein (LDL) between HYX and CTRL in either sex. However, they were significantly increased, whereas high-density lipoprotein (HDL) was significantly decreased in HFD, STZ, HFD/STZ, and HFD/STZ/HPX compared with CTRL in both sexes. The expression of *Tnf*-*α*, Il6, and* Lep* was significantly upregulated in all subgroups compared with CTRL in both sexes. STZ and HYX showed no significant differences in the expression of these genes between sexes, whereas *Tnf*-*α* and *Il6* were upregulated in male HFD, HFD/STZ, and HFD/STZ/HYX compared with females. Protein levels showed similar patterns. Combination subgroups, either in the absence or presence of hypoxia, frequently exhibited severe necrosis of endocrine components in pancreatic lobules. The combination of obesity, diabetes, and hypoxia was associated with inflammation, which was verified at the histopathological level.

## Introduction

Diabetes mellitus (DM) is a metabolic disease characterized by high levels of blood glucose (hyperglycemia) owing to an imbalance in the production of insulin (American Diabetes A, 2009; Salim, 2005; Alsaraj et al., 2009). Diabetes is a serious public health problem, and according to World Health Organization reports it has contributed to a 5% increase in premature deaths between 2000 and 2016 (Zhou et al., 2016). In 2019, diabetes ranked ninth as a leading cause of death, with nearly 1.5 million deaths (Khan et al., 2020; Muhlestein et al., 2003; Begum et al., 2014). Obesity, which is a chronic metabolic disorder affecting both adults and children, is a significant risk factor for type 2 diabetes(Lifshitz et al., 2016). Obesity is defined as the abnormal deposition of fat in adipose tissues due to prolonged overeating, low physical activity, or other inherited factors (Leitner et al., 2017). Moreover, there has been a positive correlation between obesity and type 2 diabetes, leading to what is known as diabesity (McNaughton, 2013; Chadt et al., 2018). This undoubtedly indicates that a large proportion of diabetic patients suffer from obesity, and statistics indicate that by the year 2025, the prevalence of obesity-related diabetes will double to approximately 300 million affected (Dyson, 2010). Obesity is usually associated with hypoxia (HYX), a marked decrease in blood flow, and an increase in the levels of triglycerides in the body (Hosogai et al., 2007). HYX is a situation in which the supply of oxygen is limited and its partial concentration in a tissue falls below a certain level (Pietrobon & Marincola, 2021). In a study conducted to understand the role of HYX in adipose tissues, HYX was shown to significantly contribute to the promotion of chronic inflammation, potentially causing the death of obese people (Ye et al., 2007). Exposure of male ob/ob mice (a model for diabetes and obesity (to HYX resulted in a significant increase in the expression of HYX-inducible genes (Ye et al., 2007). Mice exposed to HYX showed decreased expression of adiponectin and increased expression of inflammatory genes (He et al., 2011). Other studies indicated that HYX contributes significantly to the release of free fatty acids, which hinder glucose uptake in lipocytes by inhibiting the insulin signaling pathway and inducing cell death (Yin et al., 2009). It has also been found that middle-aged men exposed to HYX can develop type 2 diabetes (Xi, Chow & Kong, 2016). Studies have shown that the stimulation of hypoxia-inducible factor 1 occurs most commonly in the early stages of obesity as a response to systemic HYX, resulting in insulin resistance in adipocytes, as well as adipose inflammation and metabolic disorders (Lee et al., 2014; Gaspar & Velloso, 2018; Kimura et al., 2019). However, the mechanism by which HYX contributes to diabetes-related insulin resistance at the cellular and molecular level remains unknown. One hypothesis is that HYX in adipocytes might result from chronic inflammation, which contributes to insulin resistance (Trayhurn, 2005; Ye, 2009). However, there have been no clear evidence in favor of this suggestion.

The purpose of this study was to evaluate the effects of obesity, diabetes, and HYX on pancreatic tissues and serum in female and male rats, either in combination or separately, by investigating the expression of inflammatory genes and cytokines and the histopathology of pancreatic tissues. Another objective of this study was to determine whether sex differences exist (attributable to sex hormones) following the exposure of rats to obesity, HYX, diabetes, or all of them combined with chronic inflammation.

## Materials & Methods

### Animals and ethical statement

For this study, 120 male and female Wistar rats, aged 8–10 weeks and weighing 180–220 g, were obtained from the King Fahd Medical Research Center (King Abdul-Aziz University, Jeddah, Saudi Arabia). The rats were housed in metal cages in a hygienic and temperature-controlled environment (21–25 °C, humidity (50%)), with a light/dark cycle (12 h light, 12 h darkness), and had free access to normal standard diet chow and water. After one week of adaptation, the rats were divided into two experimental groups according to sex (a male and a female group). Each group was randomly divided into six subgroups ( *n* = 10 per group).

This work was performed in accordance with the National Institutes of Health’s Guide for the Care and Use of Laboratory Animals (Sciences, 2011) and the recommendations for Reporting In Vivo Experiments in Animal Research (Kilkenny et al., 2010). This study was approved by the Taif University Research Ethics Committee (No. 43-220), in agreement with the guidelines of the National Committee for Bioethics (No. HAO-02-T-105). Care was taken to reduce potential confounders during the experiment, including treatment arrangements, ensuring that the treatments were administered simultaneously. At the conclusion of the planned experiments, ketamine and xylazine were used to induce sleep in the rats to prevent them from being stressed or hurt during euthanasia.

### Experimental groups

**Figure 1:**
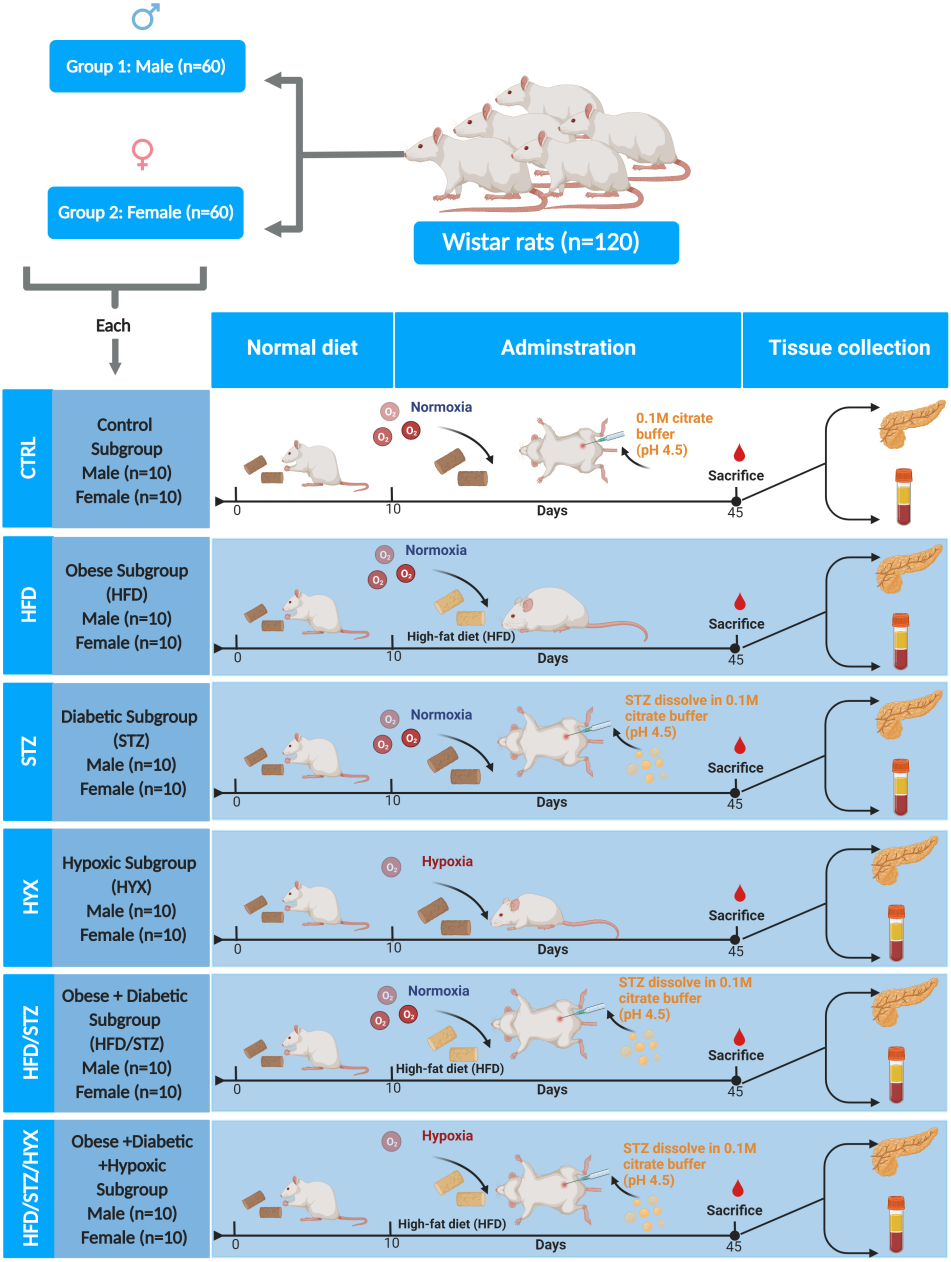
Animal groups and subgroups in this study. Abbreviations: CTRL, control subgroup; HFD, obese subgroup; STZ, diabetic subgroup; HYX, hypoxic subgroup; HFD/STZ, obese and diabetic subgroup; HFD/STZ/HYX, obese and diabetic subgroup under hypoxia. Created with BioRender.com.

Rats were divided in two groups according to sex (60 male and 60 female rats). Each group was further randomly divided into six subgroups (*n* = 10/per subgroup) by using an entirely random design shown Fig. 1.

#### Animal models

Animals were randomly divided into control and model groups. The control group was fed a standard diet and injected intraperitoneally with a single dose of 0.1 M citrate buffer (PH 4.5). The model group was further divided into the following groups:

#### Obese model

Male and female rats were fed a high-fat diet (HFD) containing 2850.00 kcal/kg of calories for 4 consecutive weeks. The composition of HFD was as follows: 25% crude protein, 14% crude fat, 35% crude fiber, 16% ash, 5% salt, 1% calcium, 0.60% phosphorus, 20.00 IU/g vitamin A, 2.20 IU/g vitamin D, 70.00 IU/kg vitamin E; added trace minerals included cobalt, copper, iodine, iron, manganese, selenium, and zinc. This diet was defined as previously described (Barrachina et al., 2020).

#### Diabetic model

Either male and female rats were fed a standard diet for 28 consecutive days and then were injected intraperitoneally with a single dose of 45 mg/kg streptozotocin (STZ (Sigma-Aldrich; Merck KGaA, Darmstadt, Germany) dissolved in 0.1 M citrate buffer (PH 4.5) to induce diabetes. If the levels of blood glucose rose beyond 18 mmol/L within 24 h of administration of STZ and remained elevated, the animals were considered diabetic. The concentration of blood glucose obtained from the clipped tip of the tail, was measured in all animals using Accu-Chek Active test strips (Roche Diabetes Care, Bella Vista, Australia). This procedure was performed according to the method previously described (Akbarzadeh et al., 2007).

#### Hypoxic model

For the duration of the experiment, both obese control and diabetic animals were housed in a glass airtight chamber and subjected to 10% O_2_ for 4 weeks. Two weeks after exposure, rats were taken from the 10% O_2_ chamber, placed in another airtight chamber, subjected to 250 ppm CO in air for only 1 h/d, and returned to the 10% O_2_ chamber. This procedure was performed as previously described (Zuckerbraun et al., 2006).

#### Obese and diabetic model

Rats were treated as described in the above sections Obese Model and Diabetic Model combined.

#### Obese, diabetic, and hypoxic model

Rats were treated as described in the above sections Obese Model, Diabetic Model, and Hypoxic Model combined.

### Determination of body weight of rats and levels of fasting blood glucose (FBG)

Each week, the weight of each rat was measured on the same day and at the same time. Rats were starved for 12 h at the end of the acclimation week, and the tip of the tail was severed using sharp scissors and gently squeezed for a drop of blood. The level of glucose in blood was measured using a glucometer (AccuChek Active; Roche Diagnostic Corporation, Mannheim, Germany). During the trial, the levels of fasting blood glucose (FBG) were measured weekly on the same day and at the same time. Calibrators provided by the manufacturer were used to calibrate the glucometer. An oral glucose tolerance test (OGTT) was performed at the end of the treatment. Glucose (2 g glucose/kg BW) was orally administered after a 12 h fast. Blood glucose levels were tested at 0, 30, 60, and 120 min after glucose administration. The OGTT-area under the curve (AUC) was calculated using the trapezoidal rule.

### Determination of insulin resistance

After the OGTT confirmed glucose tolerance, the insulin level was determined using rat-specific enzyme-linked Immune absorbent (ELISA) assay kits (Elabscience Biotechnology, Inc., Houston, TX, USA, Cat. No: E-EL-R3034). At 450 nm, the variations in absorbance were recorded. The homeostasis model evaluation of insulin resistance (HOMA-IR) was estimated using Uma’s formula (Bhandari et al., 2013).

\begin{eqnarray*}\text{HOMA-IR}=\text{Insulin}(\mu \text{U/mL})\times \text{glucose}(\text{mM})/22.5. \end{eqnarray*}

### Hematological analysis

At the end of the experiment, blood samples were collected via cardiac puncture into separate precooled heparinized containers to measure the effects of obesity and diabetes on red blood cell count, mean corpuscular volume, mean corpuscular hemoglobin concentration, and hematocrit. In addition, the number of platelets and white blood cells (all types) was also evaluated.

### Sample collection and storage

At the end of the study, rats were fasted overnight, weighed, and anesthetized using sodium pentobarbital (100 mg/kg). Pancreatic tissues were quickly removed from all subgroups, washed with ice-cold saline, one part of the tissue was placed in two mL tubes and immediately stored at −80 °C prior to RNA extraction, while the remaining tissue was fixed in 10% formalin solution prior to histopathological analysis. The serum from all subgroups was isolated and stored at −20 °C prior to biochemical analysis and cytokine assays.

### Serum lipid profile

Serum lipids were analyzed using enzymatic assay kits from NanJing JianCheng Bioengineering Inc. (Nanjing, Jiangsu, China). The levels of serum total cholesterol, serum triglycerides, high-density lipoprotein (HDL), and low-density lipoprotein (LDL) were determined using enzymatic assay kits (Cat. No. TC: A111-1, Cat. No. TG: A110-1, Cat. No. HDL: A112-1, and Cat. No. LDL: A113-1, respectively) according to the manufacturer’s protocol. All measurements were performed as previously described (Alagwu et al., 2014).

### Cytokine, and protein assays

The levels of cytokines were quantified using commercially available enzyme-linked immunosorbent assay (ELISA) kits (Elabscience Biotechnology, Inc., Houston, TX, USA). The levels of tumor necrosis factor- *α* (TNF- *α*), interleukin (IL)-10, IL6, adiponectin, and hs-CRP, were measured using enzymatic assay kits (Cat. No. E-EL-R0019, Cat. No. E-EL-R0016, Cat. No. E-EL-R0015, Cat. No. E-EL-R3002 and Cat. No. E-EL-R3012, respectively) in accordance with the manufacturer’s recommendations for each cytokine. All measurements were performed as previously described (Simpson et al., 2000).

### Gene expression analysis of pancreatic tissues

Total RNA was extracted from tiny slices of pancreatic tissues taken from animals in each subgroup using TRIzol^®^ reagent (Invitrogen, Carlsbad, CA, USA). Reverse transcription of RNA into complementary DNA (cDNA) was performed using the Access Reverse transcription-polymerase chain reaction (RT-PCR) system (Promega Corporation, Madison, WI, USA), according to the manufacturer’s instructions. Real-time PCR was performed in a PCR system using gene-specific forward and reverse primers, as specified in Table 1, to determine the expression of mRNAs in pancreatic tissues using *Actb* as a reference.

**Table 1:** Primers used for gene expression analysis of pancreatic tissues.

Target Gene	Accession ID.	Name of oligomer	Nucleotide sequence (5′ → 3′)	Reference
*Tnf-α*	ENSG00000136244	sense	5′-ACTGAACTTCGGGGTGATTG-3′	Alagwu et al. (2014)
		antisense	5′-GCTTGGTGGTTTGCTACGAC-3′	
*Il6*	ENSG00000136244	sense	5′-TGGAGTTCCGTTTCTACCTG-3′	Simpson et al. (2000)
		antisense	5′-TTCATATTGCCAGTTCTTCG-3′	
*Il10*	ENSG00000136634	sense	5′-TGCCTTCAGTCAAGTGAAGAC-3′	Hotamisligil, Shargill & Spiegelman (1993)
		antisense	5′-AAACTCATTCATGGCCTTGTA-3′	
*Lep*	ENSG00000174697	sense	5′-GCCCTATCTTTTCTATGTCC-3′	Alzamil (2020)
		antisense	5′-TCTGTGGAGTAGCCTGAAG-3′	
*Actb*	ENSG00000075624	sense	5′-CTCTTCCAGCCTTCCTTCCT-3′	Ogston & McAndrew (1964)
		antisense	5′-AAAGCCATGCCAAATGTCTC-3′	

**Notes.**

Abbreviations Tnf-α tumor necrosis factor-alpha Il6 interleukin 6 Il10 interleukin 10 Lep leptin Actb actin-beta

### Histopathological analysis of pancreatic tissues

After fixation, pancreatic tissues from animals of all subgroups were dehydrated in alcohol and xylene. Samples were dehydrated and immersed in paraffin before being cut into 3- to 5-µm-thick sections. Sections were stained with hematoxylin and eosin (H&E) at 25 °C for 5 min. Routine light microscopic examination at 40 × and 200 × magnification was used to observe morphological changes.

### Statistical analyses

SPSS 28.0.1.1 (IBM Corporation, Armonk, NY, USA) was used for statistical analysis to compare alterations in the levels of cytokines between subgroups using one-way analysis of variance (ANOVA), followed by post-hoc multiple comparisons using Duncan’s test. For the rest of the analysis, GraphPad Prism Software, LLC (version 9.3.1, La Jolla, CA, USA) was used using two-way ANOVA. For all data, *p* < 0.05 was considered statistically significant.

## Results

### Effect of STZ and HYX on rat weight

After animal modeling, we determined the levels of FBG and body weight of rats on days 0, 5, 10, 20, and 30. We found that male rats injected intraperitoneally with 45 mg/kg STZ showed decreased weight on days 5, 10, and 30 compared with those in the control subgroup, with a modeling rate of 1/6 and 0 (dead rats/group), respectively. We observed that although the administration of 45 mg/kg STZ in HFD-fed male rats caused significant weight loss on days 5, 10, and 30, the death rate and incidence of diabetes were 1/6 and 5/6, respectively (Table 2).

**Table 2:** Impact of HFD, STZ, HFD/STZ, HFD/STZ/HYX on body weight.

**Subgroups**	**Sex**	**Initial body weight**	**Body weight at the end** **of experiment**	**Changes in body weight**
CTRL	male	180.5 ± 2.10	253^bc^	+ 72.5
	female	179.75 ± 2.39	259^bc^	+ 79.5
HFD	male	181.0 ± 3.19	255.500^bc^	+ 74.5
	female	183.5 ± 2.72	249.750^cd^	+ 66.2
HYX	male	179.250 ± 2.29	284.750^a^	+ 105.5
	female	182.500	270.250^ab^	+ 87.75
STZ	male	180.0 ± 5.23	231.500^de^	+ 80
	female	183.0 ± 3.24	225.750^e^	+ 42.75
HFD/STZ	male	184 ± 1.87	242.500^cde^	+ 58
	female	185 ± 3.61	249.250^cd^	+ 64.25
HFD/STZ/HYX	male	181.750 ± 2.10	246.500^cd^	+ 64.75
	female	183 ± 3.94	246.500^cd^	+ 63.5

**Notes.**

Abbreviations CTRL control subgroup HFD obese subgroup STZ diabetic subgroup HYX hypoxic subgroup HFD/STZ obese + diabetic subgroup HFD/STZ/HYX obese + diabetic+ hypoxic subgroup

Distinct letters (a–d) in each column indicate significant differences versus CTRL.

### Oral glucose tolerence and area under the curve in experimental groups

**Figure 2:**
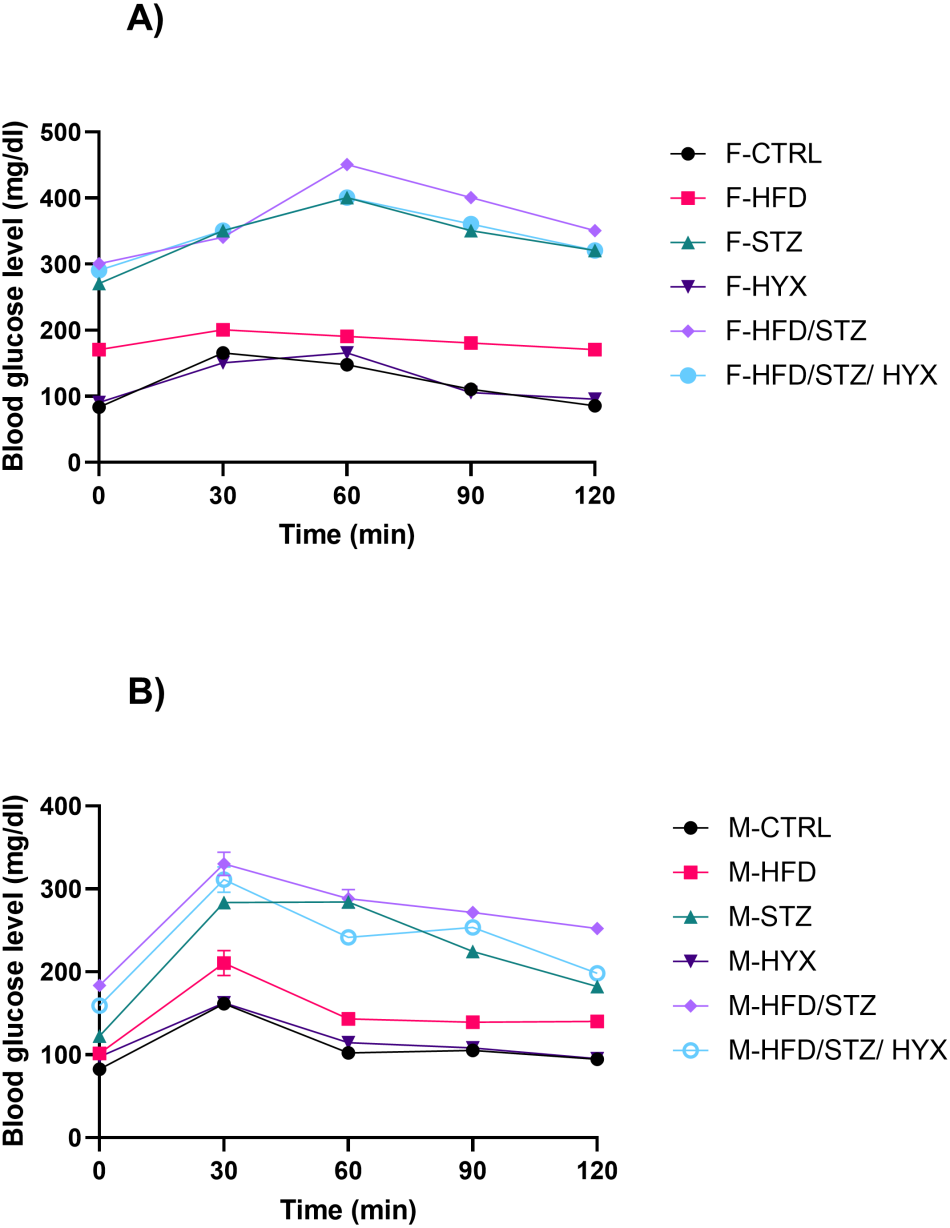
The OGTT-area under the curve (AUC) from both male and female Westar rats in the HFD, STZ, HFD/STZ, and HFD/STZ/HYX subgroups. Abbreviations: CTRL, control subgroup; HFD, obese subgroup; STZ, diabetic subgroup; HYX, hypoxic subgroup; HFD/STZ, obese and diabetic subgroup; HFD/STZ/HYX, obese and diabetic subgroup under hypoxia; ns, not significant. (A) Female group, (B) male group.

The glucose clearance and the area under the curve (AUC) of the OGTT are shown in Fig. 2. When compared to the control group, all the treatment groups’ blood glucose levels were higher at 0 and 120 min (Fig. 2). When compared to the control group, the AUC was increased in all the treatment groups.

### Insulin resistance

We used the homeostasis model assessment (HOMA) to calculate the insulin resistance index HOMA-IR, which reveals the insulin resistance based on serum insulin levels (IU/mL) and serum glucose levels (mmol/L). The current findings revealed that at the beginning of the treatment, all the diabetic rats had considerably higher HOMA-IR levels than that of the control rats (Table 3).

Table 3 shows the amounts of glucose and insulin in the blood. When compared to healthy control rats, the serum glucose levels in HFD fed rats (all groups except control and HYX) were considerably higher (*p* < 0.001). Blood insulin levels in HFD, STZ, HFD/STZ, and HFD/STZ/HYX -treated rats, either male or female, were not significantly different. Table 3 shows the HOMA-IR of all the experimental groups. When compared to the healthy control group, all animals from the HFD, STZ, HFD/STZ, and HFD/STZ/HYX rats had a significantly higher (*p* < 0.001) HOMA-IR whereas there is no significantly difference between the HYX group and the control group.

### Serum lipid profile

**Figure 3:**
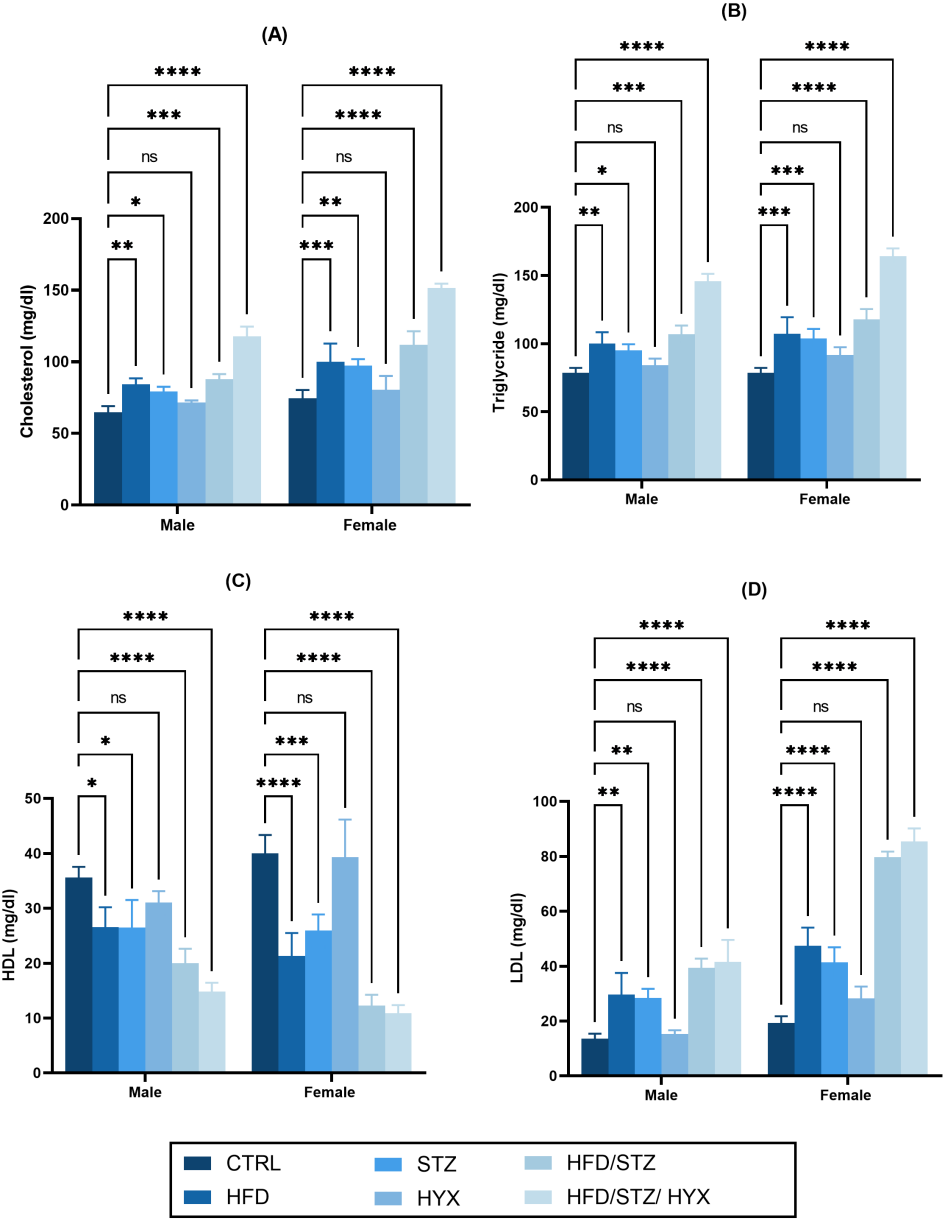
Effect of high-fat diet (HFD), streptozotocin (STZ), HFD/STZ, HFD/STZ/hypoxia (HYX) on serum lipid profile. Levels of (A) cholesterol, (B) triglycerides, (C) high-density lipoprotein (HDL), and (D) low-density lipoprotein (LDL) in both male and female Westar rats. Abbreviations: CTRL, control subgroup; HFD, obese subgroup; STZ, diabetic subgroup; HYX, hypoxic subgroup; HFD/STZ, obese + diabetic subgroup; HFD/STZ/HYX, obese + diabetic+ hypoxic subgroup; ns, not significant. Two-way analysis of variance (ANOVA) was used for data analysis. Values are expressed as the mean ± standard deviation (*n* = 10 per subgroup). At *p* < 0.05, differences were considered statistically significant.^∗^*p* < 0.05,^∗∗^*p* < 0.01,^∗∗∗^*p* < 0.001,^∗∗∗∗^*p* < 0.0001.

As shown in Figs. 3A –3D, we did not detect any significant differences in the serum levels of cholesterol, triglycerides, and LDL in the HYX subgroups compared with those in the CTRL group in either the male or female groups. However, we observed that these levels were significantly higher (*p* < 0.05) in the HFD, STZ, HFD/STZ, and HFD/STZ/HPX subgroups compared with those in the CTRL group in both the male and female groups. Additionally, we found that both male and female rats in the HFD, STZ, HFD/STZ, and HFD/STZ/HPX subgroups had significantly lower levels of HDL (*p* < 0.05) than those in the CTRL group, while the HLD levels between the HYX and CTRL subgroups showed no difference, regardless of whether male or female. From these results, it could be inferred that HFD, STZ, HFD/STZ, and HFD/STZ/HPX treatments, but not HPX treatment, could impact serum lipid profile.

**Table 3:** Baseline values of glucose homeostasis parameters in response to administration of HFD, STZ, HFD/STZ, HFD/STZ/HYX on body weight.

**Subgroups**	**Blood Glucose(mg/dl)** **Mean ± SD**		**Blood Glucose (mmol/L)** **Mean ± SD**		**Serum Insulin (µIU/ml)** **Mean ± SD**		**HOMA-IR index**	
	**Male**	**Female**	**Male**	**Female**	**Male**	**Female**	**Male**	**Female**
CTRL	95.2 ± 1.59^e^	100 ± 1.7^e^	5.28	5.25	6.32 ± 0.19^a^	6.90 ± 0.18^a^	1.483	1.61
HFD	163.0 ± 6.60^c^	166.5 ± 9.40^c^	9.05	9.22	4.70 ± 0.37^b^	3.8 ± 0.40^b^	1.890	1.55
HYX	87.0 ± 2.54^e^	82.0 ± 1.70^e^	4.83	4.55	6.60 ± 0.29^a^	6.60 ± 0.29^a^	1.440	1.334
STZ	225.0 ± 12.40^b^	220.0 ± 8.94^b^	12.5	12.20	3.86 ± 0.27^bc^	3.64 ± 0.21^b^	2.144	1.973
HFD/STZ	258.5 ± 11.3^ab^	264.0 ± 11.6^a^	14.33	14.77	3.58 ± 0.19^c^	3.72 ± 0.25^b^	2.280	2.441
HFD/STZ/HYX	284.0 ± 15.6^a^	292.5 ± 16.24^a^	15.8	16.22	4.0 ± 0.35^bc^	3.8 ± 0.34^b^	2.80	2.739

**Notes.**

Abbreviations CTRL control subgroup HFD obese subgroup STZ diabetic subgroup HYX hypoxic subgroup HFD/STZ obese + diabetic subgroup HFD/STZ/HYX obese + diabetic+ hypoxic subgroup HOMA-IR homeostasis model assessment to calculate the insulin resistance index

Distinct letters (a–d) in each column indicate significant differences versus CTRL.

### Gene expression analysis of pancreatic tissues

**Figure 4:**
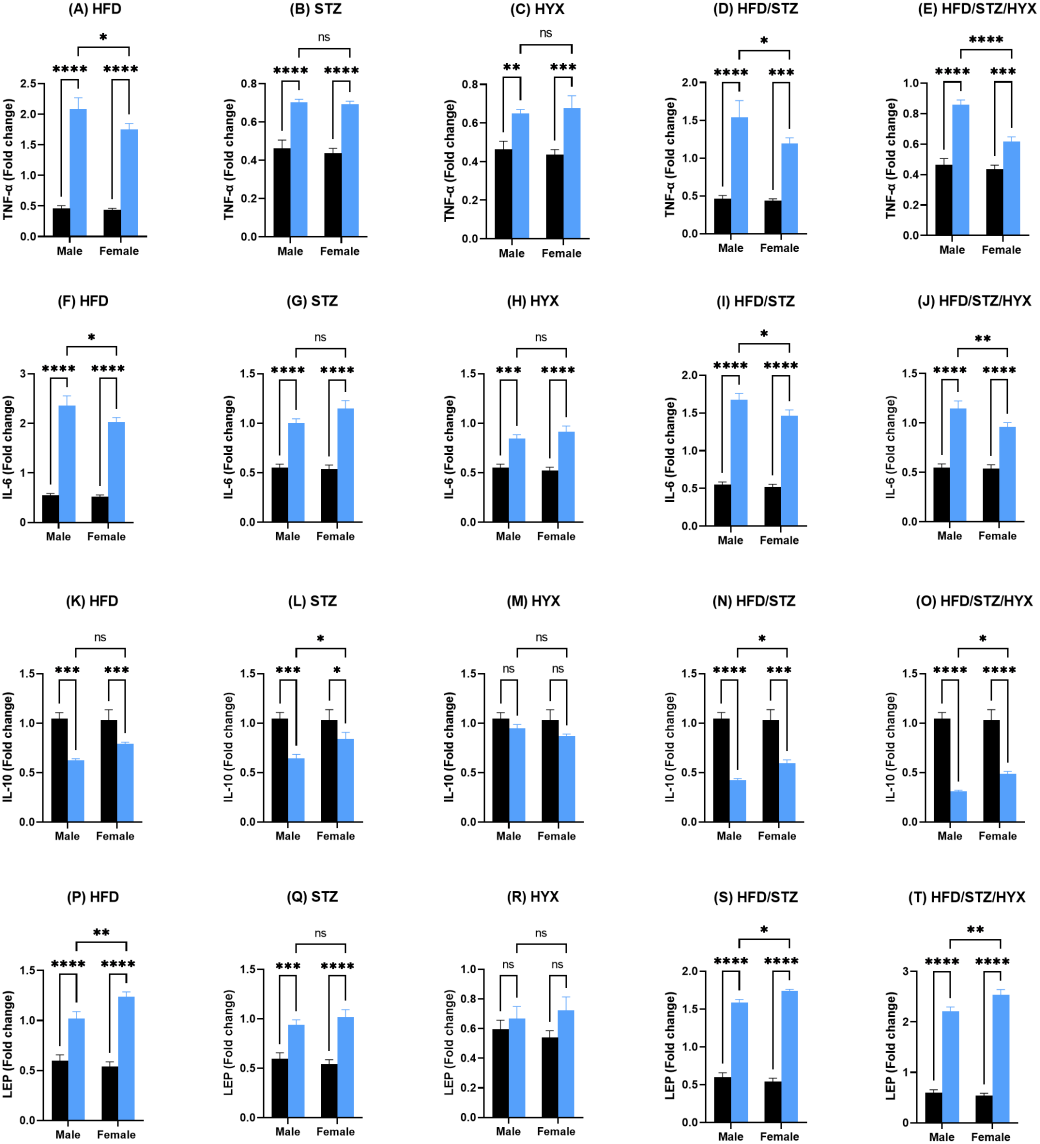
Levels of gene expression in pancreatic tissues from both male and female Westar rats in the HFD, STZ, HFD/STZ, and HFD/STZ/HYX subgroups. Abbreviations: CTRL, control subgroup; HFD, obese subgroup; STZ, diabetic subgroup; HYX, hypoxic subgroup; HFD/STZ, obese and diabetic subgroup; HFD/STZ/HYX, obese and diabetic subgroup under hypoxia; ns, not significant. Two-way ANOVA was used for data analysis. Values are expressed as the mean ± standard deviation (*n* = 10 per subgroup). At *p* < 0.05, differences were considered statistically significant.^∗^*p* < 0.05,^∗∗^*p* < 0.01,^∗∗∗^*p* < 0.001,^∗∗∗∗^*p* < 0.0001. Dark blue indicates CTRL, whereas light blue indicates other subgroups (HFD, STZ, HFD/STZ, and HFD/STZ/HYX) as indicated above each plot.

As shown in Figs. 4A –4J and 4P–4T, the mRNA expression of *Tnf- α*, *Il6*, and *Lep* was significantly upregulated (*p* < 0.05) in all subgroups compared with those in CTRL in both male and female groups. When comparing the expression of these genes between male and female rats, we found that the STZ and HYX subgroups showed no significant differences between the two sexes, whereas we observed that the expression of *Tnf- α* and *Il6* was significantly increased (*p* < 0.05) in male rats in the HFD, HFD/STZ, and HFD/STZ/HYX subgroups compared with those in female rats of the same subgroups.

Figures 4K –4O shows that the mRNA expression of *Il10* was significantly reduced (*p* < 0.05) in the HFD, STZ, HFD/STZ, and HFD/STZ/HYX subgroups compared with those in CTRL in both male and female groups. However, we detected that their expression was significantly decreased (*p* < 0.05) in male rats in the STZ, HFD/STZ, and HFD/STZ/HYX subgroups compared with those in female rats. Interestingly, we did not observe any significant differences in the expression of *Il10* mRNA in the HYX and HFD subgroups compared with that in CTRL or between male and female groups. From these results, it could be inferred that treatment with HFD, HFD/STZ, or HFD/STZ/HYX, but not STZ or HYX treatment significantly increased the expressions of *Tnf- α* and *Il6* in the male group. Furthermore, all treatments showed significant decreased expression of *IL10*.

### Alterations in levels of cytokines and proteins

**Figure 5:**
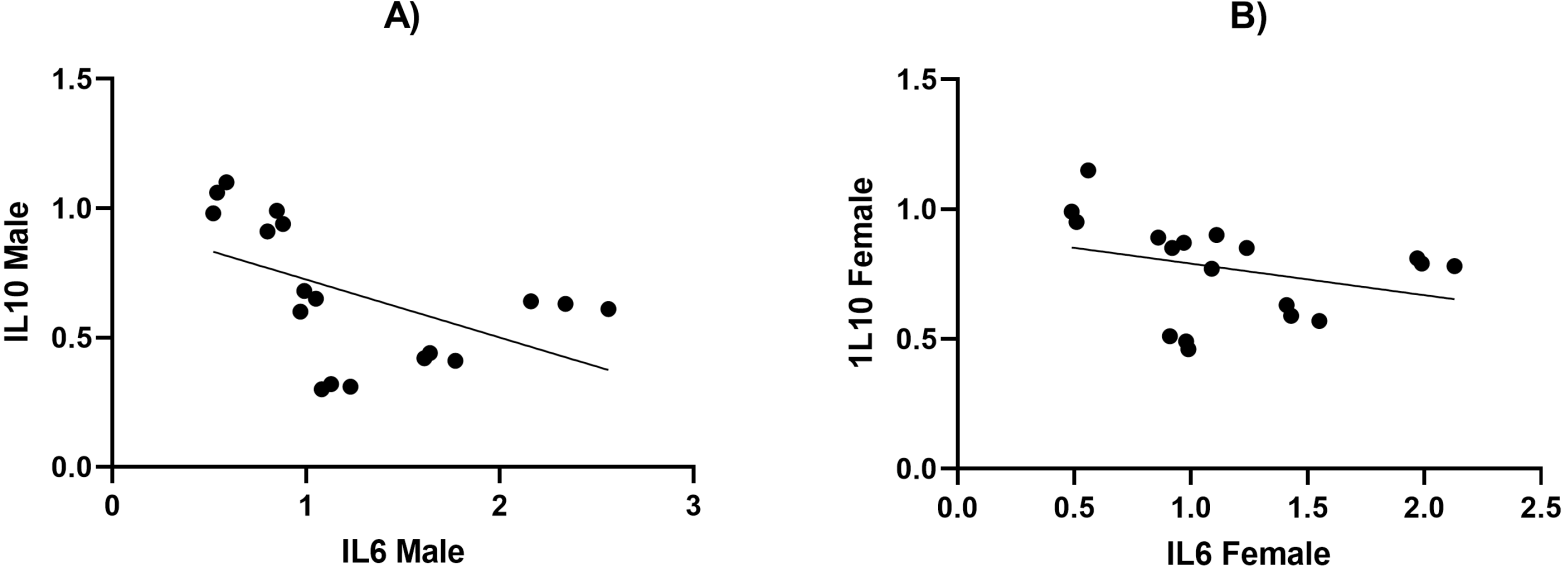
The relationship between serum levels of IL6 and IL10 in both male and female Westar rats in the HFD, STZ, HFD/STZ, and HFD/STZ/HYX subgroups. A Pearson product-moment correlation coefficient was computed to assess the relationship.

We measured the levels of TNF- *α*, IL6, IL10, and leptin using ELISA and present our results in Tables 4 and 5. We found that the secretion of TNF- *α* and hs-CRP were significantly increased (*p* < 0.05) in the male HFD/STZ and HFD/STZ/HYX subgroups, followed by the female HFD/STZ/HYX subgroup, the female STZ subgroup, both male and female HFD subgroups, and both male and female HYX subgroups in were like CTRL. The level of IL6 was statistically significant higher (*p* < 0.05) in the HFD/STZ/HYX subgroups in both males and females, then that in the HFD/STZ subgroups in both males and females, followed by HFD in both males and females more than in the other models. The leptin level was significantly higher (*p* < 0.05) in the HFD/STZ/HYX and HFD/STZ subgroups in males than in females. On the other hand, the level of IL10 and adiponectin significantly decreased (*p* <  0.05) in the HFD/STZ/HYX and HFD/STZ subgroups in males than in females and under HFD and HYPX treatments, IL10 decreased significantly (*p* < 0.05) in both sexes compared with those in control. It could be inferred that the levels of TNF- *α*, IL6, and leptin increased and that of IL 10 decreased in the HFD/STZ/HYX and HFD/STZ treatments in males, which showed a significant effect of HYX, diabetes, and obesity when combined partially or entirely with each other or anti/pro inflammatory cytokines in male rats. The relationship between the serum levels of IL6 as a pro-inflammatory factor and IL10 as an anti-inflammatory factor indicated a significantly negative correlation with *r* =  − 511 and −315 in males and females respectively; *p* < 0.05 (Figs. 5A & 5B). Increases in the serum levels of IL6 in the serum of either males or females of different treated groups were coupled with decreased serum IL10 levels. In a context characterized by an enhanced anti-inflammatory response or a decreased pro-inflammatory response, the system shifts to an active inflammatory state.

**Table 4:** Impact of HFD, STZ, HFD/STZ, HFD/STZ/HYX on levels of cytokines (TNF- *α*, IL6, IL10, and leptin).

**Subgroups**	**Gender**	**TNF- *α***	**IL6**	**IL10**	**Leptin**
CTRL	male	1.68 ± 0.08^e^	124.07 ± 0.58^f^	123.80 ± 0.60^a^	7.67 ± 0.54^f^
	female	1.79 ± 0.04^e^	126.16 ± 0.69^f^	125.23 ± 0.56^a^	6.24 ± 0.98^f^
HFD	male	16.88 ± 0.41^c^	157.17 ± 0.60^bc^	100.20 ± 0.45^d^	24.64 ± 0.59^d^
	female	19.22 ± 0.79^c^	156.26 ± 0.48^c^	99.13 ± 0.61^d^	36.84 ± 1.69^c^
HYX	male	3.40 ± 0.64^e^	125.13 ± 0.59^f^	119.06 ± 0.52^b^	6.90 ± 0.66^f^
	female	2.96 ± 0.76^e^	126.16 ± 0.23^f^	120.13 ± 0.466^b^	7.03 ± 0.94^f^
STZ	male	13.82 ± 0.70^d^	142.23 ± 0.788^e^	101.90 ± 0.57^c^	17.95 ± 0.79^e^
	female	16.27 ± 1.26^cd^	144.30 ± 0.79^d^	103.10 ± 0.66^c^	20.66 ± 1.51^e^
HFD/STZ	male	29.33 ± 1.26^a^	159.16 ± 0.66^b^	91.20 ± 075^f^	37.49 ± 0.70^b^
	female	23.61 ± 0.67^b^	158.46 ± 0.57^b^	94.36 ± 0.69^e^	41.76 ± 1.04^a^
HFD/STZ/HYX	male	29.60 ± 2.03^a^	164.93 ± 1.15^a^	90.16 ± 0.44^f^	35.31 ± 1.29^b^
	female	25.86 ± 0.83^b^	163.16 ± 0.44^a^	95.76 ± 0.39^e^	44.31 ± 2.66^a^

**Notes.**

One-way ANOVA was used for data analysis. Values are expressed as the mean ± standard deviation (*n* = 10/subgroup). At *p* < 0.05, differences were considered statistically significant. Distinct letters (a–f) in each column indicate significant differences versus CTRL.

Abbreviations TNF- *α* tumor necrosis factor- *α* IL interleukin CTRL control subgroup HFD obese subgroup STZ diabetic subgroup HYX hypoxic subgroup HFD/STZ obese + diabetic subgroup HFD/STZ/HYX obese + diabetic+ hypoxic subgroup

**Table 5:** Impact of HFD, STZ, HFD/STZ, HFD/STZ/HYX on levels of adiponectin and hs-CRP.

**Subgroups**	**Gender**	**Adiponectin** **(mg/L)**	**hs-CRP** **(ng/L)**
CTRL	male	0.716 ± 0.024^**d**^	0.50 ± 0.02^**d**^
	female	0.702 ± 0.029^**e**^	0.524 ± 0.025^**d**^
HFD	male	0.858 ± 0.030^**c**^	0.64 ± 0.022^**c**^
	female	0.898 ± 0.468^**d**^	0.62 ± 0.025^**c**^
HYX	male	0.714 ± 0.013^**d**^	0.59 ± 0.018^**c**^
	female	0.720 ± 0.021^**e**^	0.59 ± 0.033^**c**^
STZ	male	1.056 ± 0.044^**b**^	0.78 ± 0.026^**b**^
	female	1.056 ± 0.0546^**c**^	0.80 ± 0.017^**b**^
HFD/STZ	male	1.27 ± 0.033^**a**^	0.85 ± 0.016^**a**^
	female	1.284 ± 0.040^**b**^	0.88 ± 0.010^**a**^
HFD/STZ/HYX	male	1.322 ± 0.0235^**a**^	0.892 ± 0.010^**a**^
	female	1.394 ± 0.026^**a**^	0.906 ± 0.012^**a**^

**Notes.**

One-way ANOVA was used for data analysis. Values are expressed as the mean ± standard deviation (n = 10/subgroup). At *p* < 0.05, differences were considered statistically significant. Distinct letters (a–f) in each column indicate significant differences versus CTRL.

Abbreviations TNF- *α* tumor necrosis factor- *α* IL interleukin CTRL control subgroup HFD obese subgroup STZ diabetic subgroup HYX hypoxic subgroup HFD/STZ obese + diabetic subgroup HFD/STZ/HYX obese + diabetic+ hypoxic subgroup

### Histopathological examinations

**Figure 6:**
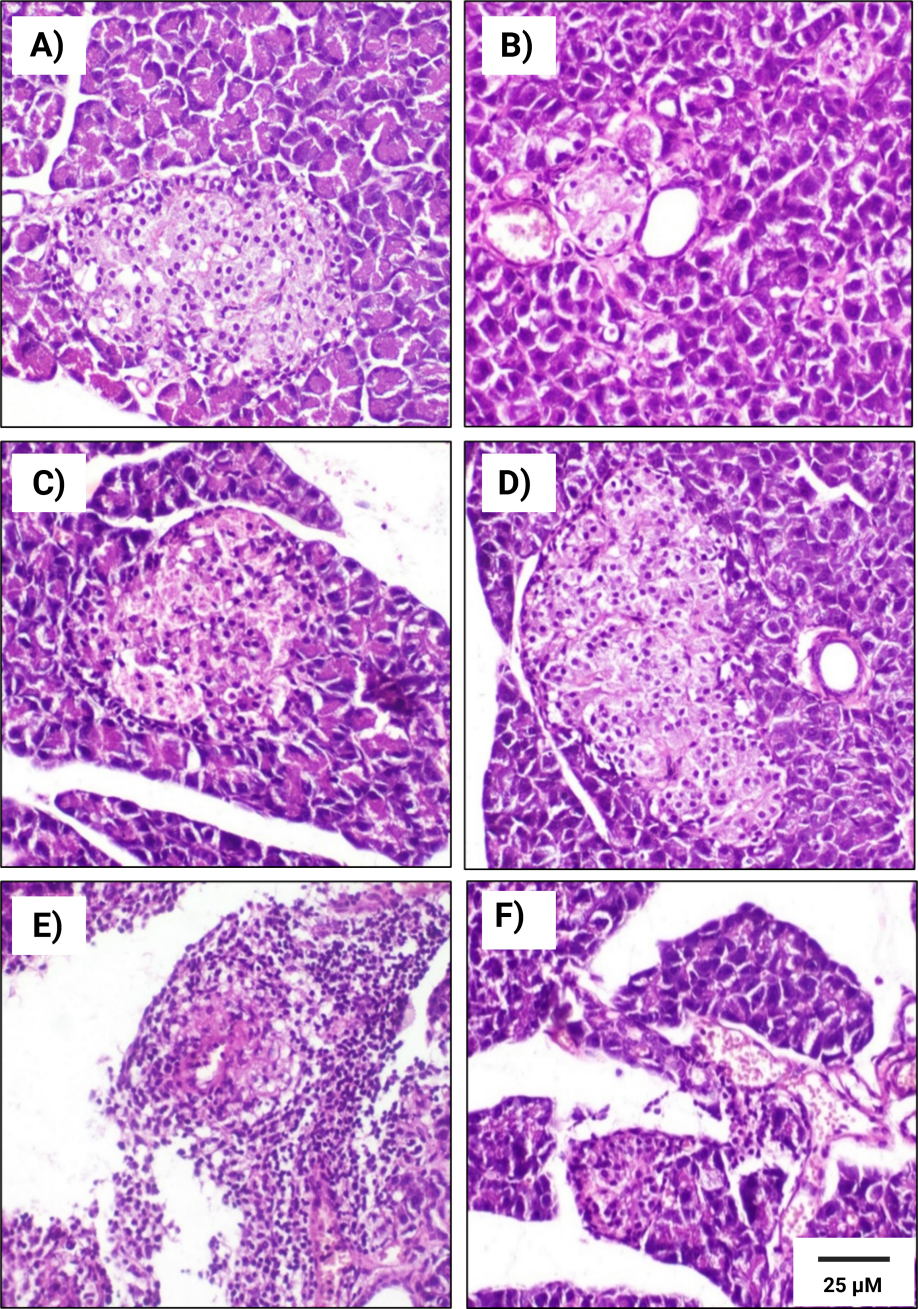
Histopathological examination of pancreatic tissues from different subgroups of Westar male rats stained with hematoxylin and eosin (H&E). (A) CTRL; control male subgroup, (B) HFD; obese male rat subgroup fed a HFD for 4 convective weeks, (C) STZ; diabetic male rat subgroup given 45 mg/kg STZ via intraperitoneal (i.p.) injection, (D) HYX; hypoxic male rat subgroup exposed to low concentration of oxygen, (E) HFD/STZ; obese and diabetic male rat subgroup (HFD subgroup administered STZ), (F) HFD/STZ/HYX; obese, diabetic, and hypoxic male rat subgroup (HFD subgroup administered STZ and subjected to hypoxia). Scale bar = 25 µm. All images are presented at a magnification of ×200.

**Figure 7:**
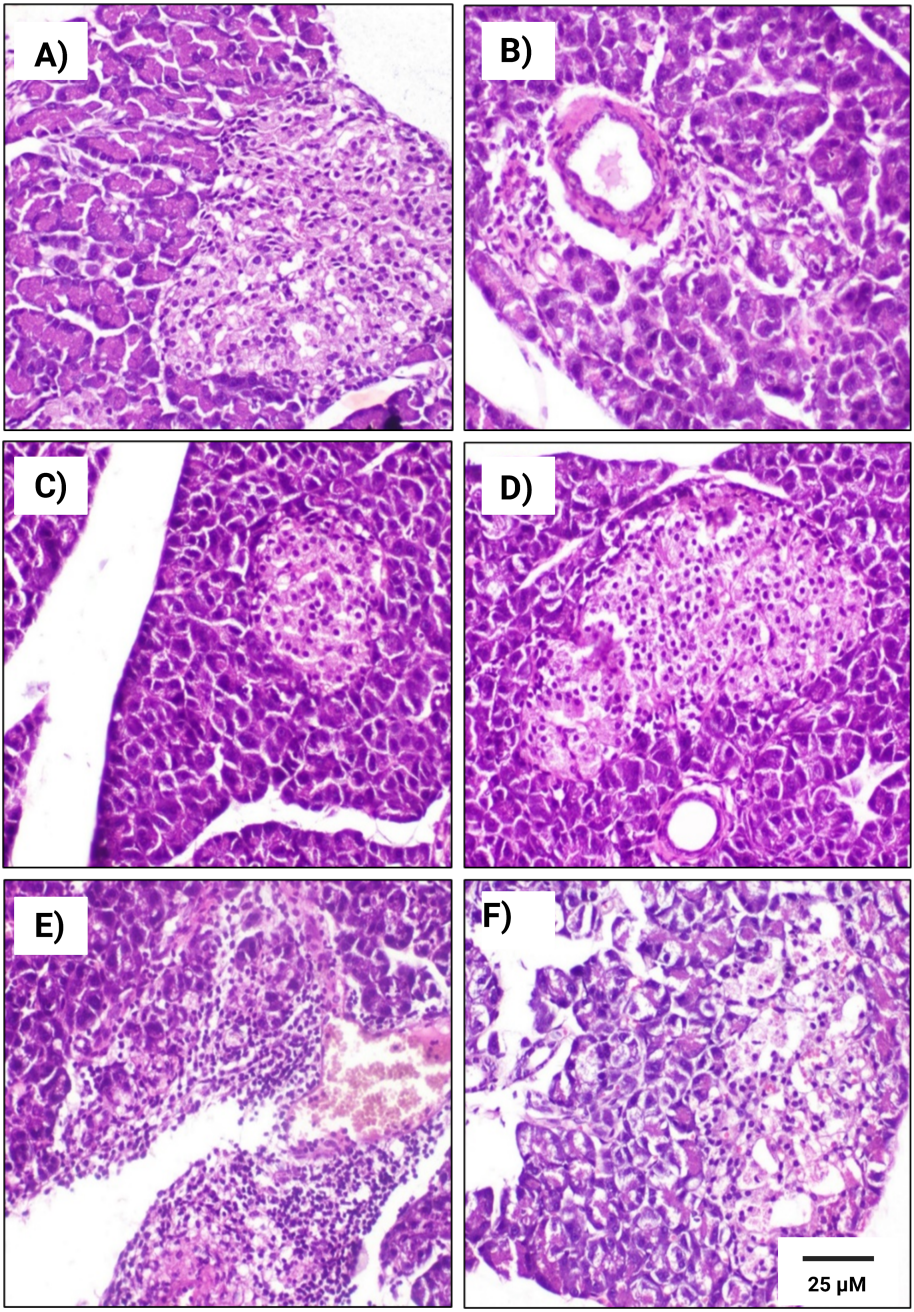
Histopathological examination of pancreatic tissues from different subgroups of Westar female rats stained with hematoxylin and eosin (H&E). (A) CTRL; control female subgroup, (B) HFD; obese female rat subgroup fed HFD for 4 convective weeks, (C) STZ; diabetic female rat subgroup administered 45 mg/kg STZ via i.p. injection, (D) HYX; hypoxic female rat subgroup exposed to low concentration of oxygen, (E) HFD/STZ; obese and diabetic female rat subgroup (HFD subgroup administered STZ), (F) HFD/STZ/HYX; obese, diabetic, and hypoxic female rat subgroup (HFD subgroup administered STZ and subjected to hypoxia). Scale bar = 25 µm. All images are presented at a magnification of ×200.

Microscopic analysis of the rat pancreatic tissues in the control subgroup, revealed normal structures in both exocrine and endocrine components of the pancreatic tissues in both male and female rats (Figs. 6A & 7A). We observed that Langerhans islets appeared normal in size and contained *β*-cells, exhibiting insignificant changes between the two sexes.

In contrast, we detected that administration of a HFD subgroup (Figs. 6B& 7B) resulted in variable numbers of small-sized ill-distinct Langerhans islets, containing few vacuolated and necrotic *β*-cells. We also noticed that some of the examined sections showed an inflammatory reaction in the peripancreatic tissue associated with congested blood vessels; however, we did not detect any significant differences between obese male or obese female rats

We found that the pancreatic tissues of diabetic rats (STZ subgroup) showed several histopathological alterations in both male and female groups. In particular, pancreatic islets showed marked necrosis and atrophy. We also observed numerous congested blood vessels among the exocrine components associated with multifocal areas of pancreatitis that were characterized by the infiltration of numerous mononuclear inflammatory cells. However, we did not detect any significant differences between male and female rats, as indicated in Table 5 and Figs. 6C & 7C.

We further observed that the pancreatic tissues of animals that were exposed to a low concentration of oxygen (HYX subgroup) were histologically normal, showing insignificant pathological changes in both the male and female groups. We found that only a few fields revealed small-sized islets and mildly dilated blood vessels compared with those in control animals, no significant differences observed between the two sexes, as shown in Table 5 and Figs. 6D & 7D.

We further observed a significant degeneration of pancreatic islets, as inferred from cellular lesions, in males and females of either the HFD/STZ subgroup or the HFD/STZ/ HYX subgroup, as evidenced by necrosis, islet atrophy, and eosinophilic material (Figs. 6E & 7E) and (6D & 7D).

**Figure 8:**
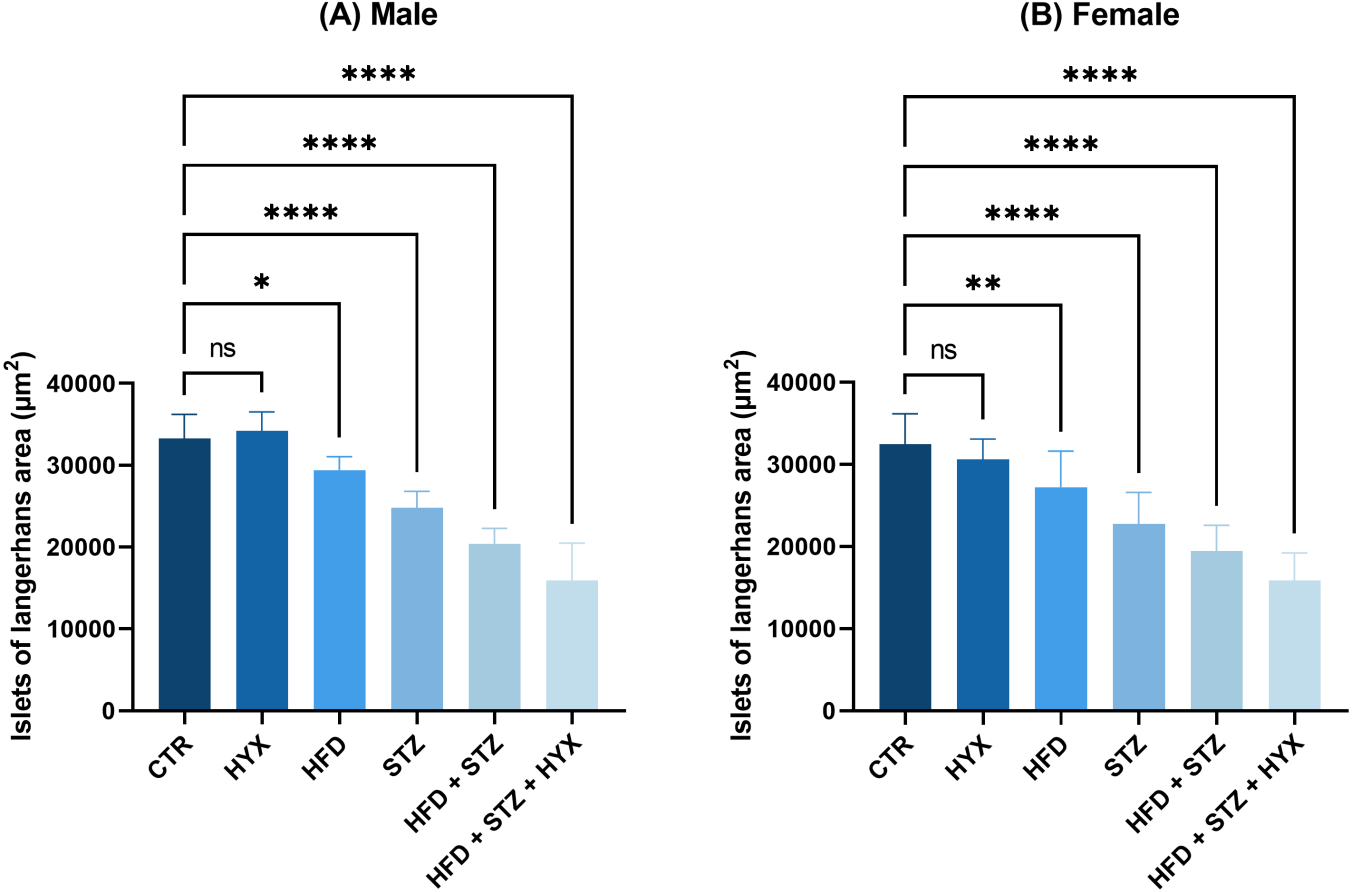
The area of Langerhans islets in the (A) male and (B) female groups. Abbreviations: CTRL, control subgroup; HFD, obese subgroup; STZ, diabetic subgroup; HYX, hypoxic subgroup; HFD/STZ, obese and diabetic subgroup; HFD/STZ/HYX, obese and diabetic subgroup under hypoxia; ns, not significant. One-way ANOVA was used for data analysis. Values are expressed as the mean ± standard deviation (*n* = 10 per group). At *p* < 0.05, differences were considered statistically significant.^∗^*p* < 0.05,^∗∗^*p* < 0.01,^∗∗∗^*p* < 0.001,^∗∗∗∗^*p* < 0.0001.

As illustrated in Table 6 and Fig. 8, the area of Langerhans islets in both male and female groups was significantly increased (*p* < 0.05) in the control subgroup when compared with each treatment except for the HFD subgroup, in which both male and female groups showed no significant change.

**Table 6:** Impact of HFD, STZ, HFD/STZ, and HFD/STZ/HYX on the area of Langerhans islets.

**Subgroups**	**Sex**	**Area of Langerhans islets (µm**^2^)
CTRL	male	33248.104^ab^
	female	32458.226^ab^
HFD	male	34178.344^a^
	female	30606.952^bc^
HYX	male	29412.665^cd^
	female	27212.204^de^
STZ	male	24788.927^ef^
	female	22765.433^f^
HFD/STZ	male	20424.722^g^
	female	19456.170^gh^
HFD/STZ/HYX	male	15941.927^i^
	female	15835.578^i^

**Notes.**

One-way ANOVA was used for data analysis. Values are expressed as the mean ± standard deviation (*n* = 10/subgroup). At *p* < 0.05, differences were considered statistically significant. Distinct letters (a–i) in each column indicate significant differences versus CTRL).

Abbreviations TNF- *α* tumor necrosis factor- *α* IL interleukin CTRL control subgroup HFD obese subgroup STZ diabetic subgroup HYX hypoxic subgroup HFD/STZ obese + diabetic subgroup HFD/STZ/HYX obese + diabetic+ hypoxic subgroup

Likewise, we detected a significant increase (*p* < 0.05) in the area of Langerhans islets in both male and female rats in the HFD/STZ and HFD/STZ/ HYX subgroups, as inferred from the above results.

## Discussion

In 1993, Hotamisiligil and colleagues performed an animal model study to understand the relationship between diabetes and inflammation. They demonstrated the role of TNF- *α* in obesity, particularly in insulin resistance and diabetes (Hotamisligil, Shargill & Spiegelman, 1993; Alzamil, 2020). Epidemiological connections between inflammation, obesity, and diabetes were also established in studies where the levels of markers of inflammation, such as IL6, C-reactive protein, plasminogen activator inhibitor-1, and fibrinogen (factor I) were elevated under these conditions (Ogston & McAndrew, 1964; Fearnley, Vincent & Chakrabarti, 1959; Kaptoge et al., 2010; Ridker et al., 1997; Duncan et al., 2003).

Chronic inflammation is associated with an increase in the amount of adipose tissue, which occurs in conjunction with the expression of several interleukins and tumor necrosis factor-*α* (Ellulu et al., 2017). A previous study examined the relationship between a HFD and the secretion of cytokines and revealed that such a diet stimulates the production of cytokines that cause inflammation and boost intestinal permeability (Liu et al., 2012).

In our study, we included a model of obese rats using both male and female animals. As was previously shown, feeding them with a HFD led to the development of an apparent obesity pattern, including a more than normal increase in weight, a significant increase in the serum levels of cholesterol, triglycerides, and LDL, and lower levels of HDL compared with those in the control group in both obese male and female groups. Although we noticed that there was a significant difference compared with those in the control group in terms of changes in gene expression and secretion of cytokines responsible for inflammation. An earlier study on obese male mice demonstrated abnormal patterns associated with sex steroid hormones, including a significant decrease in the levels of testosterone and progesterone, whereas an increase in the level of estradiol (Fan et al., 2018). Other studies have demonstrated that steroid sex hormones can play a role as a protective factor in the sense that they can regulate the activity of certain immune cells; for instance, testosterone can suppress or activate the secretion of some proinflammatory cytokines, such as interferon gamma, TNF- *α*, and IL6 (McKay & Cidlowski, 1999; Malkin et al., 2003). From another perspective, studies have shown that estrogen significantly improves immune responses at the cellular and molecular levels by activating the production of proinflammatory cytokines, such as TNF- *α*, and IL6 (Vegeto et al., 1999; Straub, 2007). Our results showed that there was a significant increase in the expression of *Lep* in both obese male and female rats compared with the control group, which was more prominent in the obese female group. Leptin is a product of the obese (ob) gene. It is synthesized and secreted by fat cells in white adipose tissues, and then attaches to and triggers its corresponding receptor, the leptin receptor (Obradovic et al., 2021). When expressed as a percentage of fat mass, women have been reported to possess higher levels of leptin than men (Hellström et al., 2000; Vettor et al., 1997; Casabiell et al., 1998).

In this study, we successfully generated a model of diabetes in both male and female rats by treating them with STZ. STZ has been shown to reliably induce diabetes in animal models with limited systemic toxicity (Marfella et al., 2002). Diabetic rats were characterized by a significant increase in their levels of FBG. This was also accompanied by an increase in weight, a significant increase in the serum levels of cholesterol, triglycerides, and LDL, whereas a decrease in the serum levels of HDL compared with those in the control group in both male and female rats. Although we noticed that there was a significant difference (*p* < 0.05) in both male and female diabetic rats compared with the control group in terms of changes in gene expression, and secretion of cytokines responsible for inflammation, including TNF- *α*, IL6, and leptin, we did not detect any sex-related differences when comparing male and female in the same subgroups. The only significant finding was the decreased expression of *IL10* in diabetic female rats compared with that in diabetic male rats, while the protein level of IL10 showed no significant difference between the sexes. This finding could be explained by the fact that the differential expression of *IL10* gene profile in the pancreatic tissue between the sexes could be distinct from the protein profile in sera, as different tissues were used in this study. It is important to state that a previous study found that the induction of diabetes in animal models might induce mutations in the *IL10* gene in some organs rather than others, resulting in alterations in the expression of IL10 in tissues (Bare et al., 2018). IL10 is an anti-inflammatory cytokine found at lower concentrations in type 2 diabetes (Naz et al., 2020). There have not been many studies comparing the levels of IL-10 between sexes; we found only one study that suggested a close association between low levels of IL10 with a metabolic syndrome in a group of women (Esposito et al., 2003).

A hypoxic model was also generated in this study but was not associate with any sex-related changes in the serum lipid profile. The gene expression of *TNF- α*, *IL6*, *IL10*, and leptin was changed in rats subjected to hypoxic conditions, but no differences were detected between the male and female groups. The idea that HYX might cause inflammation has become widespread because of research on the HYX signaling pathway (Eltzschig & Carmeliet, 2011). Inflammation that develops in response to HYX has been shown to be clinically significant (Semenza, 2007). HYX has been shown to affect the levels of mRNA and protein of pro- and anti-inflammatory cytokines, such as IL1, IL6, or IL10, modify chemokine receptors, stimulate lymphocyte production, and significantly promote subsequent signal transduction of hypoxic inflammatory responses (Li et al., 2016). Our study showed that there were no significant alterations in the mRNA expression of leptin and that of inflammatory cytokines in hypoxic male and female rats compared with those in the control, and we also did not detect any significant sex-related differences. It has been found that hypoxia promotes lipogenesis via modulating proteins involved in fatty acid absorption, production, storage, and utilization in a HIF-dependent manner (Mylonis, Simos & Paraskeva, 2019) Hypoxia promotes the absorption of extracellular fatty acids by activating the transcription factor peroxisome proliferator-activated receptors (PPARs) and increasing the expression of fatty-acid-binding proteins (FABPs) three, four, and seven (Mylonis, Simos & Paraskeva, 2019). Other investigations have demonstrated that HIF deactivation by deletion, silence, or pharmacologic inhibition can reduce lipid accumulation in several animal models and that targeting HIF expression may be a promising therapeutic strategy for metabolic diseases (Behn et al., 2007; Huang et al., 2022; Tojo et al., 2015). Furthermore, we investigated the effect of the combination of obesity and diabetes, as well as the combination of obesity, diabetes, and HYX in male and female rats, and found that these combinations altered the serum lipid profile, including increases in the serum levels of cholesterol, triglycerides, and LDL, and a reduction in the levels of HDL. Both mRNA and protein levels of pro- and anti-inflammatory cytokines were significantly altered in this subgroup in both obese and diabetic male and female rats compared with those in control, with obese and diabetic male rats, as well as obese, diabetic, and hypoxic male rats showing more significant alterations compared with obese and diabetic female rats, which could be attributed to the effect of sex hormones.

Previous studies have indicated that the plasma concentrations of proinflammatory cytokines, such as TNF *α*, and IL6 are elevated under conditions of insulin resistance in obesity and type 2 diabetes (Dandona, Aljada & Bandyopadhyay, 2004). In obesity cases, an imbalance between oxygen supply and tissue requirements might lead to increased infections in tissues, as a result of cellular infiltration, chronic low-grade systemic inflammation, and insulin resistance, further indicating the promotion of inflammation by HYX (Ye, 2009). Additionally, it is noteworthy that a boost in the levels of inflammatory cytokines is predictive of future obesity and development of diabetes (Zatterale et al., 2020). Two possible mechanisms might contribute to the pathophysiology of this inflammation. First, as obesity has been found to be a factor in oxidative stress, excessive glucose consumption might contribute to the generation of oxidative stress, which will result in inflammatory alterations. Second, elevated levels of TNF- *α* and IL-6 that have been linked with obesity and type 2 diabetes might impair insulin sensitivity by inhibiting insulin signaling pathways. This might impair the anti-inflammatory function of insulin, hence promoting inflammation.

Our histopathological results showed that in the combination subgroups, either in the absence or presence of HYX, severe necrosis of the endocrine components was frequently detected in pancreatic lobules. Vacuolation was also observed in numerous pancreatic islets. Infiltration of inflammatory cells was observed in the exocrine acini associated with infiltration of heavy mononuclear cells in adjacent surrounding adipose tissues. Finally, numerous congested blood vessels were detected in several tissue samples.

The limitations of this work include the limited sample size, the incapacity to extend these findings to humans at this time, and our inability to elucidate the molecular processes underlying the observed effects. Furthermore, Wistar rats may not be an ideal rat model for studying diabetes and obese metabolism due to their worsened metabolic effects. Rats of the Sprague-Dawley strain are regarded as a suitable model for inducing obesity by diet, as their behavior regarding excessive food consumption is comparable to that of humans and leads to weight gain and metabolic alterations in lipids; however, Wistar rats are more sensitive to diet-induced obesity than other strains, as they consume a higher proportion of high-fat food than Sprague-Dawley rats and are more susceptible to obesity (Yang et al., 2017). In addition, changes in lipid metabolism, such as fatty acid absorption and lipogenesis, and the link between genes and diet make Wistar rats more prone to diet-induced obesity (Miranda et al., 2018). The age at which high-fat feeding is initiated, the duration of high-fat feeding, and the dosages of STZ could be altered substantially, resulting in variable metabolic profiles of the animal between studies (De Moura E Dias et al., 2021). Strain and species variations must also be carefully considered when selecting a model, as various species and strains have varying susceptibilities to diabetes (King, 2012). Furthermore, Wistar rats, after administered with STZ or after high-fat fed, have been used in several studies to study diabetes and obesity because this strain can mimic the clinical symptoms found in individuals who have been diagnosed with these conditions (Skovsø, 2014; Guex et al., 2019; Kuwabara et al., 2017). However, the level to which beta-cell mass should be lowered to simulate type 2 diabetes is a possible topic of contention because if the beta-cell mass reduction is too extreme, the model could be claimed to closely match an obese type 2 diabetes model (Skovsø, 2014).

## Conclusions

Over the last few decades, our understanding of adipose tissue biology, particularly the secretory functions, has vastly increased. This breakthrough has completely altered our understanding of obesity, glucose metabolism issues, and inflammatory causation. Several cytokines have received increased attention as putative effectors in the pathophysiology and physiology of insulin resistance in people with type 2 diabetes and obesity. This study demonstrated that the combination of obesity, diabetes, and HYX was associated with the development of an inflammatory response at the mRNA and protein levels in both male and female rats; however, in male rats, there was a significant increase in the levels of TNF- *α* and IL6, and a significant decrease in IL10 compared with those in female rats. Compared with obese female rats, obese male rats were more likely to exhibit an inflammatory response. However, neither diabetes nor hypoxic conditions resulted in any sex-related differences in the inflammatory response in rats.

##  Supplemental Information

10.7717/peerj.13990/supp-1 Supplemental Information 1 Raw data: hematological analysis

10.7717/peerj.13990/supp-2 Supplemental Information 2 ARRIVE 2.0 Checklist

## Additional Information and Declarations

### Data Availability

The following information was supplied regarding data availability: The raw measurements are available in the Supplementary File.
